# Speech Processing Difficulties in Attention Deficit Hyperactivity Disorder

**DOI:** 10.3389/fpsyg.2019.01536

**Published:** 2019-07-05

**Authors:** Rina Blomberg, Henrik Danielsson, Mary Rudner, Göran B. W. Söderlund, Jerker Rönnberg

**Affiliations:** ^1^Disability Research Division, Institute for Behavioral Science and Learning, Linköping University, Linköping, Sweden; ^2^Faculty of Teacher Education Arts and Sports, Western Norway University of Applied Sciences, Sogndal, Norway

**Keywords:** attention deficit hyperactivity disorder, speech in noise, speech processing, cognitive control, working memory, auditory, adolescents

## Abstract

The large body of research that forms the ease of language understanding (ELU) model emphasizes the important contribution of cognitive processes when listening to speech in adverse conditions; however, speech-in-noise (SIN) processing is yet to be thoroughly tested in populations with cognitive deficits. The purpose of the current study was to contribute to the field in this regard by assessing SIN performance in a sample of adolescents with attention deficit hyperactivity disorder (ADHD) and comparing results with age-matched controls. This population was chosen because core symptoms of ADHD include developmental deficits in cognitive control and working memory capacity and because these top-down processes are thought to reach maturity during adolescence in individuals with typical development. The study utilized natural language sentence materials under experimental conditions that manipulated the dependency on cognitive mechanisms in varying degrees. In addition, participants were tested on cognitive capacity measures of complex working memory-span, selective attention, and lexical access. Primary findings were in support of the ELU-model. Age was shown to significantly covary with SIN performance, and after controlling for age, ADHD participants demonstrated greater difficulty than controls with the experimental manipulations. In addition, overall SIN performance was strongly predicted by individual differences in cognitive capacity. Taken together, the results highlight the general disadvantage persons with deficient cognitive capacity have when attending to speech in typically noisy listening environments. Furthermore, the consistently poorer performance observed in the ADHD group suggests that auditory processing tasks designed to tax attention and working memory capacity may prove to be beneficial clinical instruments when diagnosing ADHD.

## Introduction

Children generally have greater difficulties than adults listening to speech in adverse conditions. Maturation of the auditory system over the first decade is undoubtedly associated with age-related improvement in speech understanding in the presence of noise; however, given that both linguistic and cognitive abilities develop simultaneously with auditory abilities, it is unlikely that maturation of the auditory system alone can account for the widely observed performance differences in children that extend well into adolescence (for review, see Litovsky, 2015). The ease of language understanding (ELU) model (Rönnberg et al., 2013, 2019), which underpins the main theoretical perspective in the current research, provides an overarching account of the role that auditory, linguistic, and cognitive mechanisms play in relation to speech understanding. The model is built upon of large body of research, which demonstrates that effective speech-understanding in noise (SIN) requires complex interactions between both bottom-up and top-down processing (for review, see Stenfelt and Rönnberg, 2009).

Bottom-up processing proceeds automatically and encompasses the auditory system’s ability to parse and decode the phonetic content of speech and to transiently match/compare that content in working memory with pre-existing lexical and semantic representations in long-term memory. If the phonetic content is clearly discernable from the noise and easily matched to pre-existing linguistic representations, then the brain can implicitly comprehend the speech in a rapid, unimpeded fashion. However, if this transient matching process renders in too much error, for example, when noise grossly degrades the speech or when linguistic knowledge is deficient, then top-down processes must resolve the decoding task (Rönnberg et al., 2010). Top-down processing recruits cognitive control mechanisms such as attention, inhibition, and recall of pre-existing knowledge about the language and available contextual cues, in order to explicitly discern the speech content from the noise in working memory. The extent in which a child has developed the capacity to utilize and integrate both bottom-up and top-down processing greatly determines how well they cope in adverse listening conditions (McCreery et al., 2017). Accordingly, the ELU model emphasizes the functional importance of working memory capacity and cognitive control mechanisms when bottom-up processing is undermined. Indeed, a widely replicated finding in the literature is that measures of working memory capacity, cognitive load, attention, and inhibition correlate with individual differences in SIN (Rönnberg et al., 2013). Despite this extensive support for the functional role of working memory capacity and cognitive control mechanisms, the predictions of the ELU-model are yet to be thoroughly tested in populations with deficits in top-down processing. The purpose of the current study, therefore, was to contribute to the field in this regard.

Attention deficit hyperactive disorder (ADHD) is a neurocognitive condition in which hallmark symptoms manifest as developmental deficits in cognitive control (Mueller et al., 2017; Rubia, 2018). Because patients generally have difficulties regulating attention, inhibition, and maintaining information in working memory (for review, see Pievsky and McGrath, 2018), ADHD presents a prime case for which to study the ELU models’ top-down component. The current study tested predictions of the ELU model in a sample of Swedish adolescents (11–18 years). The primary goal was to test the general hypothesis that SIN should be more difficult for adolescents with ADHD than their age-matched counterparts due to a compromised cognitive control system and inefficient working memory capacity (Pievsky and McGrath, 2018; Rubia, 2018). Secondary aims assessed competing hypotheses regarding the effects certain types of noise have on ADHD.

The experiment was designed to examine the ELU model’s top-down component by utilizing conditions that hampered bottom-up processing and increased the dependency on cognitive control mechanisms and working memory capacity in varying degrees. To this end, participant’s SIN abilities for two types of signal quality were assessed under three different masking conditions using age-appropriate sentence materials from the Swedish hearing-in-noise task (HINT-C; Hällgren et al., 2006; Hjertman, 2011). In addition, participants were tested on cognitive measures of complex working memory-span, selective attention, and lexical access. We hypothesized (**H**_**1**_) that ADHD participants would demonstrate inferior performance to their age-matched controls on all cognitive measures due to developmental deficits in this domain (Takács et al., 2014; Pievsky and McGrath, 2018). Furthermore, in line with the ELU model, it was hypothesized (**H**_**2**_) that ADHD participants would require on average higher signal-to-noise ratios (SNRs) than controls for efficient SIN because of the increased processing demand background noise places upon top-down processes. Additionally, we expected (**H**_**3**_) individual differences in the cognitive measures to predict overall listening performance in noise.

The signal-quality conditions comprised distortion-free clear (CLR) speech and 12-channel noise-vocoded (NV) speech. NV speech is an acoustic distortion that limits the temporal fine structure and spectral detail of speech but preserves the temporal envelope and is highly intelligible in quiet. Importantly, the effect of the distortion involves greater reliance upon top-down processes than CLR speech to understand in the presence of noise (Rosemann et al., 2017), so we predicted (**H**_**4**_) participants would require higher SNRs to understand NV speech than CLR speech. For noise comparisons, participants’ speech recognition was evaluated under fluctuating (amplitude-modulated) speech-shaped noise (SSN), two-talker babble (2BAB), and stationary white noise (WN) because these three types of maskers have been shown to place differential demands on top-down processes (Rönnberg et al., 2010).

Multi-talker babble places high demands on cognitive control and working memory processes – particularly when the babble contains only a few speakers (≤4) and is perceptually similar to the speech signal (Rosen et al., 2013). Moreover, multi-talker masking affects age groups differently depending upon the predictability of the speech signal, which has been associated with developmental differences in the top-down capacity to inhibit attention to irrelevant speech, and utilizes pre-existing knowledge to infer the content of the babble-masked signal in working memory (Buss et al., 2016). Because HINT sentences provide sufficient contextual support to facilitate prediction of final words (e.g., *Grandma eats porridge every day*), we hypothesized (**H**_**5**_) that participants’ age would covary with SIN performance and (**H**_**6**_) that participants would generally require higher SNRs in conditions where the masker was perceptually similar to the speech signal. In the specific case of CLR signal-quality conditions, 2BAB was perceptually more similar to the speech signal than the two energetic noise conditions (i.e., WN and SSN). Hence, we expected higher SNRs for CLR speech in babble than energetic noise. Because listening in the amplitude dips of fluctuating noise generally requires more cognitive effort than stationary noise (Rönnberg et al., 2010), we also expected listening in WN to result in the lowest SNRs across CLR-speech comparisons. In the case of NV speech, the signal distortion likened a harsh, robotic whisper, which made it perceptually more similar to the energetic noise conditions than the audibly distinct, non-distorted 2BAB. It was therefore hypothesized that listening to the NV speech in energetic noise would result in higher SNRs than in 2BAB, and in particular, the fluctuating SSN should yield the highest SNRs due to the increased demand on top-down processes. The pressing question, however, was how adolescents with ADHD would perform under these specific manipulations compared to their age-matched counterparts. We assessed three competing outcomes based upon previous reports in the ADHD literature.

One potential outcome **(O1)** was that all three maskers would negatively impact ADHD participants’ top-down processing such that the ADHD group would require higher SNRs than controls in all experimental conditions. A similar finding was reported by Geffner et al. (1996) in ADHD children (6–12 years) that tested SIN in three types of maskers: stationary WN, cafeteria noise, and a single talker. Both ADHD and controls demonstrated excellent speech-recognition skills in quiet, however, in noise, the ADHD group was inferior to controls across all masking conditions. Pillsbury et al. (1995) tested school-aged children (8–16 years) and also found stationary SSN to impact speech-recognition thresholds more negatively in ADHD participants than age-matched controls; furthermore, overall SIN performance covaried significantly with age.

A second possible outcome **(O2)** was that in certain conditions, the ADHD group would compensate for task difficulty by exerting more cognitive effort than controls. This is a commonly reported phenomenon in the ADHD literature, which typically manifests as equivalent performance to controls on a behavioral level but significantly different task-related activation patterns at the neural level (e.g., Suskauer et al., 2008; Biehl et al., 2016). Behavioral measures that are sensitive to individual differences in cognitive capacity are also used to reveal underlying differences in cognitive strategies, even though task-related differences are not observed at the group level. For instance, Michalek et al. (2014) tested predictions of the ELU model using 5-talker babble in a sample of young adults with and without ADHD. Although they did not observe a significant group difference in SIN performance (without the aid of visual cues), they found a significant relationship between measures of working memory capacity and SIN ability in their noisiest condition (0 dB SNR) for the ADHD group. The author’s concluded, in support of the ELU-model, that ADHD participants were relying more heavily upon working memory in this condition than controls in order to maintain a commensurate level of performance. When applying this prediction to the current study, potentially, ADHD adolescents would have sufficient spare capacity under less demanding conditions to exert compensatory strategies (cf. Rudner and Lunner, 2014). As such, we would not observe a significant group difference in SNRs when listening to CLR speech in energetic noise (hypothesized to be the least cognitively demanding, see above); but individual performance would still correlate highly with the cognitive measures. Logically, it follows from this outcome that the high cognitive demand of the NV speech would usurp ADHD participants’ limited cognitive capacity making it difficult to compensate at levels equivalent to controls. We should therefore observe significantly poorer performance in the ADHD group across all maskers for the NV condition.

Interestingly, one line of research offered a third potential outcome **(O3)** that is contrary to the predictions of the ELU model. This perspective suggests that ADHD is differentially affected by stationary stochastic noise (e.g., WN) and that the cognitive control system can *benefit* from this kind of noise stimulation. In their moderate brain arousal (MBA) model, Sikström and Söderlund (2007) argue that low levels of tonic dopamine (implicated in ADHD) place the brain in a poorly aroused state, which directly affects top-down processing due to an inability to filter out irrelevant sensory information, i.e., an abundance of sensory driven bottom-up input. Low levels of tonic dopamine yield inattention (e.g. Volkow et al., 2009). Stimulating the brain’s auditory system with an optimal level of external stationary noise is thought to increase arousal and subsequently enable efficient cognitive control through the mechanism of stochastic resonance where a certain amount of noise can facilitate neural transmission and interact with the target signal and thus make it stronger (for definition, see McDonnell and Ward, 2011). Söderlund and Jobs (2016) tested the MBA model’s predictions for SIN in a sample of schoolboys (9–10 years, ADHD vs. controls). They presented sentence materials in stationary SSN at a fixed level of 65 dB SPL. Under these conditions, the ADHD group’s resulting SNR for speech recognition was shown to be on par with that of controls. Because performance differences in quiet were significant between groups, the non-significant effect in noise was interpreted as an indication of stochastic resonance. The authors concluded that participants with ADHD can benefit from noise in the context of speech recognition, provided the noise is energetic and stochastic, and presented binaurally at a moderate intensity of 65–80 dB SPL. In the current experiment, the speech signal was fixed at 70 dB, and the initial SNR was 0 dB (presented binaurally). From the perspective of the MBA model, the WN should have a beneficial effect on ADHD participants’ speech perception; hence, they should perform at least as well as controls in both the CLR and NV conditions.

O1 and O2 present outcomes that are consistent with the ELU model’s prediction that noise masking impacts speech processing by placing increased demands upon top-down processing. The third prediction (O3) from the MBA model, however, conflicts with the ELU model in which it does not predict a negative effect of WN masking in ADHD but rather a contributory benefit to speech perception. It should be noted that although O3 predicted that ADHD participants would perform on par with controls, this finding would not be conclusive evidence that participants benefited from the noise (because participants may have exerted more effort as in O2 above). However, a replication of the finding in Söderlund and Jobs (2016) that applies even in the NV condition would certainly warrant further consideration for the MBA model. Still, more convincing evidence for a beneficial effect of WN would be revealed if ADHD participants demonstrated efficient performance at significantly lower SNRs than controls.

## Materials and Methods

### Participants

The study was approved by the Regional Ethics Committee in Linköping, Sweden (Dnr 2016/169-31). Participants volunteered for the study through advertisements posted in schools, clinics, and online social media platforms. Volunteers who met the inclusion criteria were recruited for the study regardless of where they resided in Sweden. For both groups, the principal inclusion criteria were an age requirement of 11–18 years; Swedish as a first language; and the ability to make an informed decision about participation on one’s own accord. In addition, the inclusion requirements for the ADHD group were:

1. A formal diagnosis of ADHD according to Swedish interdisciplinary assessment standards (Granholm et al., 2016) operating under the framework of DSM-5 ADHD criteria.
2. Children who were not treating their ADHD symptoms with prescription medication or children who were taking central stimulants but agreed to a 24-h washout from medication immediately prior to the day of participation.

**Figure 1:**
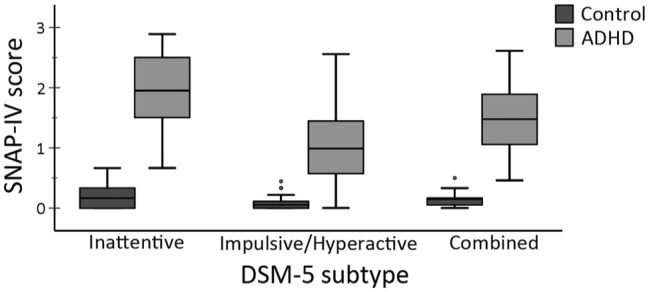
SNAP-IV parental ratings for ADHD symptoms per DSM-5 subtype and group. SNAP-IV scores range from 0 (no symptoms) to 3 (highly frequent symptoms). Boxplots represent min/max, interquartile range, and median.

At the time of data analysis, participants were excluded if the pure-tone audiogram indicated non-normal hearing acuity (see below), or they were unable to discern the speech materials in quiet at a threshold ≤60 dB SPL (the average sound-pressure level of conversational speech at a distance of 1 m). Because experimental assumptions required that the presence of ADHD symptoms were absent in the control group, control children were additionally excluded if their parent’s ratings on the SNAP-IV ADHD rating scale (see below) exceeded the 90th percentile for symptom scores pertaining to any DSM-5 subtype. In all, a total of 42 participants were recruited, four of which were excluded from analysis: three because speech-reception thresholds (SRTs) in quiet exceeded 60 dB and one control participant because SNAP-IV ratings indicated a high level of inattentiveness. The remaining 38 participants consisted of 22 controls (*M*_age_ = 16, SD_age_ = 2.6, males = 8), and 16 ADHD participants (*M*_age_ = 14.6, SD_age_ = 2.2, males = 10). See Table 1 and Figure 1 for further information regarding participants’ hearing, cognition, and symptom scores.

**Table 1:** Group statistics for cognitive capacity and hearing in quiet tasks.

Task	Measure	Control	ADHD	*F*(1, 36)	*ω*^2^
**Cognitive capacity**					
Reading span	% Recall	0.51 (0.14)	0.37 (0.13)	10.1**	0.19
SIC span	% Recall	0.62 (0.13)	0.41 (0.13)	24.6***	0.38
d2 test	Std. score	107 (8.1)	96 (9.5)	17.3***	0.30
Lexical decision	Rate corr./s	1.0 (0.2)	0.8 (0.2)	7.0*	0.14
**Hearing in quiet**					
Pure-tone avg.	dB	−0.7 (7.9)	4.1 (5.7)	4.3	0.08
CLR-SRT	dB	43 (4.2)	47 (5.0)	8.4**	0.16
NV-SRT	dB	51 (6.8)	56 (3.9)	5.5*	0.11

Table shows group means (standard deviations), F-statistics, and effect sizes (ω^2^) for significant results (*p < 0.05, **p < 0.01, ***p < 0.001).

### Procedure

#### Sound Materials

Sound materials were presented to participants using closed Sennheiser HD 205 headphones from a Windows laptop computer (64-bit OS, Intel® Core™ i7-4700MQ @ 2.4 GHz). All auditory stimuli were created in MATLAB and calibrated for the presentation hardware by the Department for Technical Audiology at the Linköping University. The speech materials were suitable for children (Hjertman, 2011) and consisted of phonemically balanced Swedish sentences that were 3–7 words in length (the shortest sentence had a duration of 1.6 s and consisted of three words; the longest sentence had a duration of 3.4 s and consisted of four words). NV sentences were generated by first dividing the frequency range of non-distorted sentences into 12 logarithmically spaced channels before applying the amplitude envelope from each channel to band-limited noise within the same frequency band. Each band of noise was then recombined to create the noise NV sentences, which were adjusted with root-mean-squared equalization to match the sound levels of the original sentences. The WN masker consisted of a sound file with equally distributed frequencies (0–8 Hz). 2BAB was created by mixing the soundtracks of two native Swedish speakers (one male and one female) reading from a Swedish newspaper. The fluctuating SSN was constructed by modulating the SSN of the target speech with the low-pass filtered (<32 Hz) instantaneous amplitude of the 2BAB.

#### Test Procedure

Participants were tested in a quiet room/location at their place/town of residence (e.g., a secluded room in the home or the local library). All tests, with exception for the d2 test of attention (see below), were installed on a laptop computer and ran from a MATLAB platform. During auditory tests, participants were told to place themselves in a comfortable position and to concentrate upon the sound stimulus from the headphones. During cognitive tests, participants viewed the laptop computer screen on a table at a comfortable distance in front of them and responded to the tasks using left/right mouse buttons when required. The d2 test of attention was a pen-and-paper task, which participants performed seated at a table. Because the entire experimental session took circa 75 min to complete the test leader encouraged participants to take short breaks between tests if needed.

#### Auditory Tests

The auditory tests were administered in the following order: pure-tone audiogram, CLR speech in quiet, NV speech in quiet, then HINT. For all speech understanding tasks, sentence trials were marked as accurate if the participant could orally recite the entire sentence without error. The detailed procedure for each of these auditory measures is outlined below.

##### Hearing in Quiet

Participants were screened for normal hearing thresholds (<20 dB HL for the octave frequencies 0.25–8 kHz) using the standard, revised Hughson-Westlake approach on a MATLAB-based audiometer (Cooke, 1999). Pure-tone averages were derived by calculating the grand mean of all octave frequency thresholds in both ears. Participants were also screened for the ability to understand both CLR and NV speech in quiet. Resulting SRTs represented the minimal level at which participants could correctly repeat the sentences two out of three times and were obtained using a descending approach from 70 dB SPL (−5, +2 dB). Together, the hearing in quiet tasks took circa 15 min to complete.

##### Hearing in Noise

For the HINT test (Hällgren et al., 2006), CLR and NV sentence lists (20 unique sentences in each) were presented in WN, 2BAB, and fluctuating SSN. The speech signal was held at a constant of 70 dB SPL and the noise varied adaptively in steps of 2 dB from the initial SNR of 0 dB SPL. The resulting outcome measure was the mean SNR for 50% correctly repeated sentences and was estimated from the last 16 sentences in each list per experimental condition (the first four sentences were used as practice trials). The order of conditions (masker type per signal quality) was counterbalanced using a diagram balanced Latin squares protocol, and the entire test procedure had a duration of approximately 30–35 min.

#### Cognitive Measures

The extent of ADHD symptoms in participants was obtained from parents by way of the SNAP-IV rating scale, and cognitive control was assessed through Swedish versions of three different tasks: reading span, size-comparison span (SIC span), and d2 test of attention. In addition, participants’ efficiency in accessing lexical information in long-term memory was measured with a lexical decision task. The cognitive test battery was administered after completion of the HINT task in the following order: d2 test, reading span, SIC span, and lexical decision. The procedure for each of these cognitive measures is detailed below.

##### SNAP-IV ADHD Scale

The SNAP-IV parent/teacher questionnaire (Swanson et al., 2012) is a neuropsychological 4-point scale (0 = *not at all*, 3 = *very much*) designed to assess the extent a child expresses symptoms of hyperactivity/impulsivity and inattention associated with ADHD. Parental ratings were obtained with the 33-item Swedish version (Dunerfeldt et al., 2010), and scores were calculated on the first 18 items corresponding to the ADHD subtypes specified in DSM-5: inattention (items 1–9), hyperactivity/impulsivity (items 10–18), and combined inattention hyperactivity (items 1–18). In clinical settings, parental scores exceeding the 95th percentile for each subtype are considered diagnostically relevant (attentive disorder ≥ 1.78 points, hyperactivity disorder ≥ 1.44 points, combined attentive and hyperactive disorder ≥ 1.67 points).

##### d2 Test of Attention

Proficiency in selective attention was assessed using the 4 min d2 test of attention (Brickenkamp et al., 2010). The d2 test is a standardized neuropsychological test that requires participants to mark (pen stroke on paper) under time constraints, target characters embedded in strings of distractor characters (12 lines of 57 characters, 25–26 targets in each; 20 s allowed per line). The resulting score used in this study was the total number marked target characters minus the total number of commission and omission errors. The scores were transformed into standardized scores (min = 70, max = 130 points) according to age norms for which a higher score corresponded to greater proficiency in selective attention.

##### Reading Span

The reading span test (Rönnberg et al., 1989) presented participants with eight unique lists of three-word sentences in increasing length (2 × lists of 2, 3, 4, and 5 sentences). Sentences within each list were presented one word at a time (interstimulus interval = 0.8 ms), and participants were required to both remember and classify (*yes/no* button press) each presented sentence as sensical or absurd (e.g., *Dogs bark loudly* in contrast to *Fish climb trees*). After each list presentation, participants were asked to orally recall either the first or the last word (determined pseudo randomly) of each sentence in the list. The resulting reading span measure was the % of correctly recalled words for correctly classified sentences (max = 28) and represents participants’ capacity to maintain and process information in working memory (Rönnberg et al., 2016). The test took participants on average 8 min to complete.

##### Size-Comparison Span

The SIC-span test (Sörqvist et al., 2010) presented 10 unique lists of target nouns together with distracting noun pairs of increasing length (2 × lists of 2, 3, 4, 5, and 6 items). All nouns in each list belonged to the same taxonomic category (mammals, fruit, etc.). Within each list, the task was to first answer a question (*yes/no* button press) about the relative size of the noun pairs (e.g., are *raspberries* bigger than *watermelons*?) and to remember a target noun (e.g., *banana*) presented immediately after each size comparison. Because noun pairs belong to the same category as the target noun and must be processed in working memory, they are considered a semantic distraction to the memory task. At the end of each list, participants were asked to orally recall the target nouns. The resulting SIC-span measure was the % of correctly recalled targets corresponding to each correctly answered comparison (max = 40) and represents participants’ capacity to maintain and process relevant information and to inhibit competing semantic information in working memory. The entire task had a duration of approximately 10 min.

##### Lexical Decision

A lexical decision task (Holmer et al., 2016) was used to examine lexical access efficiency. The task was to determine as quickly and as accurately as possible (*yes*/*no* button press) if a string of three letters equated a real Swedish word or not. The test consisted of 40 items divided into three lists: 10 pseudowords (e.g., *wox*), 10 nonwords (e.g., *wxa*), and 20 real words of high familiarity (e.g., *wax*); the presentation order was counterbalanced over all three lists. The 40-item list took all participants less than a minute to classify. The dependent measure was calculated by dividing the total number of correct responses per participant by the amount of time (seconds) spent responding on all trials; the resulting lexical decision score represented the number of correctly classified words per second (Woltz and Was, 2006).

### Data Analysis

#### Missing Data

In total, 6.7% of values were missing from the dataset of cognitive measures (listed above), which arose in most part from technical/procedural difficulties but also due to the occasional decision from a participant to terminate a task mid-session (often related to fatigue). When all variables were entered into a missing value analysis (implemented in IBM SPSS 25 statistical software), Little’s MCAR test (Little and Rubin, 2002) revealed that the values were missing completely at random, *χ*^2^(10) = 7.3, *p* = 0.70. However when age was entered as a predictor, Little’s MCAR test was significant, *χ*^2^(13) = 403.2, *p* < 0.001, indicating that the pattern of missing values was not *completely* random but instead randomly distributed across age, a pattern termed “missing at random” (MAR; see Acock, 2005 for details). The expectation maximization method was therefore used to impute (iterations = 5,000) the missing items because it is suitable for datasets with MAR patterns and is a robust imputation method that produces a single, complete dataset with less bias then listwise/pairwise deletion or mean-replacement methods (Acock, 2005). Additionally, a single value was missing from the dataset of auditory tests due to a participant’s decision to abstain from completing a particular hearing-in-noise condition. For consistency, the missing value was also replaced using the expectation maximization single-imputation method (iterations = 5,000). For all proceeding statistical analyses, the complete dataset with imputed values was used.

#### Statistical Analyses

All analyses were undertaken in IBM SPSS. Comparisons between groups in age, hearing thresholds in quiet (pure-tone averages, SRT), and cognitive measures were analyzed with a one-way ANOVA. The distribution of gender between groups was compared with a Fisher’s exact test. To assess HINT performance, a mixed repeated-measures ANCOVA was undertaken and included one grouping factor (ADHD, controls); two repeated measures: signal quality (CLR, NV) and masker type (WN, SSN, 2BAB); and age (mean centered) as the covariate. Bonferroni-corrected planned comparisons investigated differences between maskers (WN vs. SSN vs. 2BAB) for both CLR and NV speech and also group differences for each masker per speech-quality condition in accordance with predictions (H_6_; O1–O3). Estimates of means and standard deviations (adjusted for the covariate) were reported, and partial-eta squared ($\eta_{p}^{2})$ was used to assess effect size. A two-step hierarchical multiple regression analysis was conducted to examine the relationship between overall HINT performance and cognitive capacity (H_3_). Because the variables pertaining to working memory capacity (reading span and SIC span) and selective attention (d2 score) are thought to tap into the same underlying top-down construct, principle components analysis (PCA) was used to derive a single latent predictor which we denoted *cognitive control*. Similarly, in order to assess if differences in hearing acuity were also predictive of HINT outcomes, a single-latent predictor representing *baseline acuity* was derived from participants’ pure-tone averages and SRTs-in-quiet using PCA. The first step in the regression analysis included the hearing-in-quiet predictor, the second step added predictors corresponding to cognitive capacity: cognitive control and lexical access efficacy (lexical decision score) using the forced entry method (both predictors entered into the model in one step and in order of decreasing tolerance). The dependent measure was the grand mean over all HINT conditions for each participant. Model assumptions were assessed statistically.

## Results

### Group Comparisons

By design, there were no significant differences between groups in age *F*(1, 36) = 3.0, *p* = 0.09, *ω*^2^ = 0.05 or gender proportions (*p* = 0.19, Fisher’s exact test). In addition, the presence of ADHD symptoms in the control group was negligible (all scores < 0.7) according to parental SNAP-IV ratings (Figure 1). Table 1 reports the group means, standard deviations, and results of the one-way ANOVA for the cognitive capacity measures and hearing thresholds in quiet. As hypothesized (H_1_), the ADHD group’s performance in working memory capacity, selective attention, and lexical access efficacy was significantly inferior to the control group.

The between-group comparison for pure-tone averages fell slightly under the threshold of significance (*p* = 0.05), but for speech in quiet the ADHD group required on average a 4.5 dB increase in both signal-quality conditions to accurately repeat the sentences. To explore this difference in thresholds more closely, a Pearson’s correlation analysis (*two-tailed*) was undertaken in which the relationship between hearing thresholds and cognitive capacity was investigated. Results revealed that individual differences in pure-tone averages were moderately associated with the variance in both CLR (*r* = 0.63, *p* < 0.001) and NV (*r* = 0.48, *p* = 0.002) SRTs. In addition, pure-tone averages were negatively correlated with selective attention scores (*r* = −0.40, *p* = 0.013) demonstrating that detecting a tone in quiet involves attentional mechanisms. No other correlations between hearing thresholds and cognitive capacity variables were observed.

### HINT-Analysis

**Figure 2:**
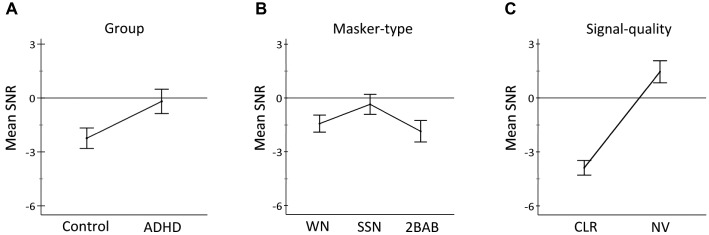
Graphs show significant main effects for **(A)** group (controls vs. ADHD), **(B)** masker type (WN: white noise; SSN: fluctuating speech-shaped noise; 2BAB: two-talker babble), and **(C)** signal-quality manipulation (CLR: clear vs. NV: noise-vocoded speech). Positive and negative estimated marginal means of SNRs resemble a reduction (<70 dB) and increase (>70 dB) in noise levels, respectively. Error bars represent 95% confidence intervals.

Results from the repeated-measures ANCOVA indicated that the covariate age was a strong predictor of SNRs, *F*(1, 35) = 8.9, *p* = 0.01, $\eta_{p}^{2}$ = 0.20 (H_5_). After controlling for age, there was a significant main effect of group, *F*(1, 35) = 21.3, *p* < 0.001, $\eta_{p}^{2}$ = 0.38, confirming (H_2_) that the ADHD group had greater difficulty than controls at understanding speech in noise (Figure 2A). There was also a main effect of masker type, *F*(2, 70) = 14.9, *p* < 0.001, $\eta_{p}^{2}$ = 0.30 in which SNRs were lowest for 2BAB, closely followed by WN, and highest for fluctuating SSN (Figure 2B). In addition, a main effect of signal quality *F*(1, 35) = 366.2, *p* < 0.001, $\eta_{p}^{2}$ = 0.91 confirmed (H_4_) that NV speech was more difficult than CLR speech to understand in the presence of noise (Figure 2C).

**Figure 3:**
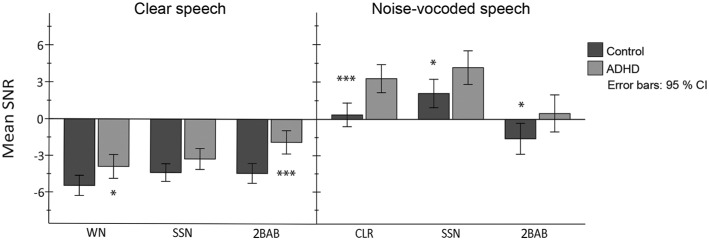
Results of between- and within-group comparisons per noise condition (WN: white noise; SSN: fluctuating speech-shaped noise; 2BAB: two-talker babble) and signal-quality manipulation (clear vs. noise-vocoded speech). Positive and negative estimated marginal means of SNRs resemble a reduction (<70 dB) and increase (>70 dB) in noise levels, respectively (the speech-signal was held at a constant of 70 dB). Asterisks indicate significant between-group differences for each masker type per signal-quality condition (^*^p < 0.05, ^***^p < 0.001, Bonferroni corrected).

A significant interaction between masker type and signal quality was also observed, *F*(2, 70) = 23.6, *p* < 0.001, $\eta_{p}^{2}$ = 0.40 suggesting that performance associated with differences in signal quality was also differentially affected by masker type (Figure 3). Bonferroni-adjusted statistical comparisons verified (H_6_) that participants’ SNRs were differentially affected as a function of the perceptual overlap between masker and signal. As hypothesized, 2BAB was shown to have the highest masking effect on CLR speech, whereas WN had the least (2BAB > WN, *p* < 0.001; 2BAB > SSN, ns; SSN > WN, ns) and for NV speech, fluctuating SSN had the greatest masking effect and 2BAB had the least (SSN > 2BAB, *p* < 0.001; SSN > WN, *p* < 0.05; WN > 2BAB, *p* < 0.001). No other interactions were significant; instead, response profiles differed only in elevation with the ADHD group performing consistently poorer than controls.

Results from the Bonferroni corrected group comparisons revealed that the ADHD group required significantly higher SNRs than controls in all maskers (as predicted in O1) except for SSN, where a statistical group difference was not apparent for the CLR-speech condition (Figure 3). To investigate if this non-significant finding corresponded with the predictions from O2, a Pearson’s correlation analysis (*two-tailed*) was undertaken to determine if there was a relationship between individual differences in SNRs and cognitive capacity for this condition. Complex working memory (reading span, *r* = −0.46, *p* = 0.004; SIC span *r* = −0.40, *p* = 0.013) and selective attention (*r* = −0.42, *p* = 0.009) were negatively correlated with SNRs. There was no significant association, however, between SNRs and lexical access efficacy (*r* = −0.30, *p* = 0.063). This finding is in line with the prediction from O2 and suggests that participants with less efficient cognitive control found the task’s conditions more taxing even though efficiency in lexical processing was similar across participants for this condition.

### Regression Analysis

#### Latent Regressors

Two principal component analyses were used to generate two latent regressors corresponding to cognitive control and baseline hearing acuity. The cognitive control regressor was derived from reading span, SIC span and d2 test variables, all of which were standardized by way of *z*-transformation prior to component extraction. Bartlett’s test of sphericity indicated that these three variables were sufficiently correlated, *χ*^2^(3) = 33.1, *p* < 0.001; and Kaiser-Meyer-Olkin’s test indicated that the sampling adequacy = 0.68 was reasonable. The extracted component had an eigenvalue = 2.1 and explained 70.5% of the variance. The baseline acuity regressor was compiled from the hearing-in-quiet measures, which were scaled (*z-*transform) prior to component extraction: pure-tone averages, CLR-SRT, and NV-SRT. Bartlett’s test indicated sufficiently large correlations between the individual measures, *χ*^2^(3) = 32,4, *p* < 0.001; and Kaiser-Meyer-Olkin’s score = 0.69, which indicated adequate sampling adequacy. The resulting component had an eigenvalue = 2.1 and explained 70.5% of the variance. For both the cognitive control and baseline acuity components, the regression method was used to compute the factor scores for each participant.

#### Model Assumptions

Pearson’s cross correlation analysis (Table 2) was used to assess the assumption of linearity and no perfect collinearity. All regressors had a significant linear association with the outcome variable (HINT). Although the predictor pertaining to cognitive control was moderately correlated with the other two regressors, variance inflation factors did not indicate high collinearity (VIF > 5) among the predictors (VIF_max_ = 1.3). A non-significant Shapiro-Wilk test, *W*(38) = 0.98, *p* = 0.698; indicated that the distribution of residuals did not deviate from the assumption of normality and a non-significant Koenker’s BP test (LM = 0.75, *p* = 0.862) supported the assumption of homoscedastic residual variance. Cook’s distance measure also indicated that no single value had an excessive influence (*D* > 1) over either regression model as a whole (*D*_max_ = 0.15).

**Table 2:** Cross correlations (Pearson’s *r, two-tailed*) between the outcome (HINT) and regressor variables (cognitive control, lexical access, and baseline acuity).

		1	2	3	4
1	HINT	–			
2	Cognitive control	−0.67***	–		
3	Lexical access	−0.68***	0.42**	–	
4	Baseline acuity	0.44**	−0.32*	−0.18	–

Asterisks indicate statistically significant correlation coefficients (*p < 0.05, **p < 0.01, ***p < 0.001).

#### Regression Results

Table 3 reports the parameters for the two regression models. The first model indicated that baseline hearing acuity was a significant predictor of outcomes, *F*(1, 36) = 8.7, *p* = 0.006, and accounted for 19% of the variance in participant’s general SIN ability (adjusted *R*^2^ = 0.17). The addition of the two cognitive predictors in the second model (cognitive control and lexical access efficacy) made a significant improvement in predictive power (see Table 3) and the resulting omnibus model, *F*(3, 34) = 25.7, *p* < 0.001 explained 69% of the variance in overall HINT performance (adjusted *R*^2^ = 0.67). All predictors made significant contributions to the final model, of which cognitive control and lexical access predictors made contributions of relatively equal importance to model outcomes (*β* = −0.41 and −0.47, respectively). Taken together, these results robustly support the hypothesis (H_3_) that individual differences in cognitive capacity predict speech understanding performance under adverse listening conditions.

**Table 3:** Two-step regression model for HINT outcomes and associated contributions of baseline hearing acuity, cognitive control, and lexical access efficacy.

	Model 1			Model 2		
	B	SE B	β	B	SE B	β
Constant	3.1	1.0		3.0	1.0	
Baseline hearing acuity	0.8	0.3	0.44**	0.4	0.2	0.23*
Cognitive control				−0.8	0.2	−0.41**
Lexical access				−4.6	1.0	−0.47***
*R*^2^		0.19			0.69	
*∆R*^2^					0.50	
*F* for change in *R*^2^					27.8***	

Asterisks indicate statistically significant coefficients (*p < 0.05, **p < 0.01, ***p < 0.001).

## Discussion

The purpose of the current study was to test the ELU model’s (Rönnberg et al., 2013) general hypothesis that SIN should be more difficult for adolescents with ADHD than age-matched controls due to their deficient capacity to regulate attention and inhibition and maintain information in working memory. The experiment tested two types of signal quality (CLR speech vs. NV speech) under three different masking conditions (WN, amplitude-modulated SSN, and 2BAB) using HINT sentence materials (Hällgren et al., 2006). These experimental manipulations were chosen because they have been shown to place differential demands upon top-down processing (Stenfelt and Rönnberg, 2009; Rönnberg et al., 2010, 2019). Participants were also assessed on cognitive capacity measures of complex working memory span, selective attention, and lexical access. Primary findings were in support of the ELU model. Results corresponding to the specific manipulations are discussed in detail below.

### Effect of Age

The study was designed to test the ELU model’s cognitive (i.e., top-down) component associated with SIN processing. To minimalize confounds of auditory development, the experimental sample included adolescents between 11 and 18 years because maturity of the auditory system typically proceeds over the first decade of life (Moore and Linthicum, 2007). In addition, the experiment controlled for differences in linguistic development by utilizing natural language speech materials appropriate for the age group being tested (Hjertman, 2011).

Cognitive processes are known to mature during adolescence (Luna, 2009; Peverill et al., 2016) and in support of the ELU-model’s predictions, studies have shown that younger teens require higher SNRs than older teens/adults when listening to speech in conditions that place demands on top-down processes (Stuart, 2008; Jacobi et al., 2017). Accordingly, age was shown in the current study to significantly covary with HINT performance as anticipated. After controlling for age, significant main effects of masker type, signal quality, and group remained, which was indicative that the experimental conditions were placing differential demands upon individual participants’ cognitive capacity.

### Effect of Signal Quality

Participants found listening to NV speech in noise more challenging than CLR speech and ADHD participants required higher SNRs than controls in all NV conditions. These results were expected because NV speech in noise is known to increase reliance upon top-down processing (Rosemann et al., 2017), and hence, performance under such conditions should be impaired (relative to age-matched controls) in ADHD. Interestingly, ADHD participants’ perception of both CLR and NV speech was shown to be differential to controls even in quiet. It is, however, not uncommon in the literature that individuals with ADHD show impairments on central auditory processing tasks even though they have normal peripheral hearing (e.g., Gascon et al., 1986; Lanzetta-Valdo et al., 2016; Fostick, 2017). Indeed, a subject of some controversy is whether central auditory processing disorder is a distinguishable diagnosis from ADHD (Moss and Sheiffele, 1994; Riccio and Hynd, 1996).

Central auditory processing tasks assess skills such as auditory closure, binaural integration, and temporal order judgment, all of which are necessary for efficient speech perception. In the current study, the variance in SRTs in quiet was not correlated with any of the cognitive capacity measures but instead associated with individual differences in pure-tone averages. Individual differences in pure-tone averages were, however, associated with attentional performance. Clearly, listening to a signal in decreasing levels of intensity involves attentional mechanisms; albeit for speech discrimination in quiet, behavioral measures that tap into domain-general selective attention may not be sufficiently sensitive to explain individual differences in performance. Speculatively, central auditory processing tasks may be more appropriate measures at detecting attentional impairments associated with fine-grained auditory discrimination in quiet. The poorer performance observed in the ADHD group in quiet may indeed be representative of the central auditory processing impairments that are commonly associated with ADHD in the literature.

### Effect of Noise

Performance associated with differences in signal quality was also differentially affected by masker type, and the absence of group interactions demonstrated that the different types of maskers affected participants’ listening performance in a similar fashion. This finding robustly supports the ELU model’s predictions about the varying degrees these specific maskers tax top-down processes.

Masking can have both beneficial and negative effects on attentional performance. Positive masking effects arise when a masker drowns out the negative impact of other competing noises – for instance, the continuous hum from a ventilation system in an office may drown out potentially distracting voices in the surrounding environment and enable one to concentrate more efficiently to information in the immediate environment. Negative masking effects arise when the attended signal is masked by the noise. The current study has only investigated the effects of masking upon the speech signal, thus in the discussion that follows, the term “masking effects” refers to the latter case of a masked signal.

#### Two-Talker Babble

A variety of studies have shown that the adverse effects of informational masking are relative to the degree of perceptual similarity the masker has with the target speech (Rosen et al., 2013). In the current study, the 2BAB masker consisted of non-distorted speech from two native Swedish speakers, and the target speech was either clear (CLR-condition) or distorted (NV-condition). Hence, the CLR speech was perceptually more similar to the non-distorted babble than the NV speech. When compared to the energetic maskers, the predicted difficulty associated with greater perceptual similarity between masker and target was evident. CLR speech was more challenging in 2BAB than in fluctuating SSN and static WN. Conversely, when the target speech was noise vocoded, the non-distorted 2BAB was shown to have a lesser masking effect than the other two maskers.

Interestingly, this confirmed effect of perceptual similarity held true only when comparing maskers. When signal-quality conditions are compared, the NV speech, despite being more perceptually distinct than the non-distorted chatter, proved to be more challenging in 2BAB to participants than CLR speech in 2BAB (95% CI of mean difference in SNR = 1.5, 3.7). Furthermore, in both signal-quality manipulations for 2BAB, ADHD participants’ performance was significantly inferior to controls. This result suggests that the process of both ignoring competing speech and attending to degraded speech is greatly more taxing on cognitive processes than suppressing the disturbance of competing speakers when listening to a target of high-acoustic quality. NV speech is frequently used in the literature to simulate cochlear implant processing, which, due to technological and biological limitations, results in a signal with high spectrotemporal degradation. The implication of this finding highlights the disadvantage in processing load cochlear implant users face in daily listening conditions (c.f. Overstreet and Hoen, 2018). Furthermore, in line with the ELU-model, this finding emphasizes that the coping advantage normal-hearing persons with high cognitive capacity have when processing degraded speech (e.g., from a loudspeaker, or poor phone connection) in acoustically crowded environments.

#### Fluctuating Speech-Shaped Noise

When listening to NV speech, fluctuating SSN had the greatest masking effect of all three maskers and ADHD participants needed higher SNRs than controls for speech understanding to be effective. The fluctuating masker together with CLR-speech did not result in a significant group difference in SNRs; however, further inspection confirmed that better task performance in this condition corresponded with proficiency in cognitive control. Stuart (2008) researched the effects of fluctuating maskers in children and adolescents in comparison with adults. General findings indicated that developing children were able to benefit from listening in the amplitude dips of the noise, but the process was thought to place greater reliance upon cognitive capacity. Thus, younger children (<14 years) tend to require higher SNRs than older adolescents/adults in fluctuating maskers, which coincide with their ongoing development of cognitive skills. Because the ADHD participants, in accord with their diagnosis, demonstrated poorer capacity on measures of attention and complex working memory, our finding, after controlling for the effects of age, is indicative that the ADHD group may have been using more cognitive effort than controls to solve the task of piecing together in working memory sparse glimpses of speech amidst the noise.

This finding aligns with the work of Michalek et al. (2014) who tested the predictions of the ELU model in adults with ADHD. The authors compared performance to controls in SIN tasks both with and without visual cues. The masker consisted of 5-talker babble. Studies have shown that when there are more than four background talkers, the temporal fine structure, and envelope of the masker starts to resemble fluctuating SSN (Rosen et al., 2013). In their auditory only condition (i.e., without the aid of visual cues), Michalek et al. (2014) found their ADHD participants performed as well as controls in the nosiest (SNR = 0) condition and performance for the ADHD group correlated with measures of working memory capacity. The authors speculated under the framework of the ELU model that ADHD participants were exerting more cognitive effort in order to maintain a commensurate level of performance under the noisiest condition. Following through on this perspective, the significant difference between groups for the NV condition in our study is suggestive that the additional demands of the distortion rendered the ADHD group with insufficient capacity to solve the task at equivalent SNRs to controls. Thus, how accurately SIN is understood is intricately related to the individual’s available capacity to compensate for the degraded auditory processing (cf. Rudner and Lunner, 2014).

#### White Noise

As discussed above, when it comes to masking effects, a masker is more challenging to speech perception the more similar it is to the acoustic qualities of the signal. The results confirmed this general pattern. Although the continuous WN was perceptually similar to the NV speech, WN masking effects were not as severe as the fluctuating masker in which the spectrotemporal variance was even more similar to the NV signal. In addition, the expected pattern of masking effects was similar for both groups, albeit the ADHD group needed more favorable SNRs than controls across maskers for NV speech. In CLR-speech conditions, the WN was expected to have the least masking effect, which was also evident in our results. Unlike the fluctuating masker, however, there was a significant group difference in which the control group coped much better in higher levels of WN than the ADHD group.

One outcome explored in this study was whether continuous auditory WN could benefit the ADHD group as postulated by the MBA model (Sikström and Söderlund, 2007). Our results are not in favor of the MBA model (Sikström and Söderlund, 2007), which predicted that ADHD participants would perform at least as well as controls in WN due to the mechanism of stochastic resonance (McDonnell and Ward, 2011). Stochastic resonance applies when the fidelity or the amplitude of an output signal from a suboptimal nonlinear system is enhanced by stochastic stationary noise (e.g., WN or stationary SSN), which improves the system’s representation of the input signal. In the case of auditory processing, two types of stochastic resonance have been observed (Zeng et al., 2000; Behnam and Zeng, 2003): (1) threshold stochastic resonance wherein the signal is presented at levels below detection threshold, and the addition of noise amplifies the signal enabling it to be detected by the auditory system and (2) suprathreshold stochastic resonance where the signal is presented above the detectable threshold, and the addition of noise (at some optimal level) improves the fidelity of the signal and enhances fine temporal signal discrimination. The mechanism of stochastic resonance, however, is not yet fully understood, and whether the phenomenon of noise benefit acts merely at the perceptual level or at a top-down level, through the integration of neural activity from many sources, remains to be determined. If the latter is the case, positive effects in persons with ADHD should not be observed in SIN tasks that place lesser demands on cognitive processing. Our materials, however, were shown to involve cognitive processing in which proficiency on the HINT task improved as a function of cognitive capacity across participants. Furthermore, we specifically varied the cognitive demands of the task by manipulating signal quality, effectively enabling us to compare the effects of the maskers under two different levels load. We saw, however, no indication of noise benefit in the ADHD group in either load condition. This leaves open the question as to whether the mechanism of stochastic resonance can enhance signal processing at higher levels than the perceptual system in the context of auditory processing.

In a pilot study, Söderlund and Jobs (2016) tested the MBA model’s predictions for SIN in a sample of schoolboys (9–10 years) with and without ADHD. The authors hypothesized that children with ADHD would demonstrate poorer SRTs in quiet than the control group, but in stationary SSN, the ADHD group would benefit from the noise (*via* suprathreshold stochastic resonance) and the variance in SRTs between groups would be neutralized. Their statistical results confirmed this hypothesized interaction of noise level (quiet vs. 65 dB WN) by group. Söderlund and Jobs (2016) concluded that in order for the beneficial effects of suprathreshold stochastic resonance to occur in the context of speech processing, the noise should be presented binaurally and at an intensity of 65–80 dB SPL. Our experiment presented all auditory materials binaurally and held the speech signal at a fixed 70 dB; the initial SNR was 0 dB and adjusted adaptively according to participants’ responses. Thus, our experimental conditions should have been sufficient to induce the mechanism of suprathreshold stochastic resonance, particularly in the NV condition in which the processing demands upon the cognitive system were increased. However, we did not observe any interactions with the WN masker. The ADHD group’s performance was significantly poorer to controls both in quiet and WN and for both signal-quality conditions. Hence, we did not observe evidence to suggest a WN benefit in the ADHD group given our experimental manipulations.

One possible reason for the discrepancy between studies is the differences in the speech materials used. HINT sentence materials differ from the Hagerman sentences (Hagerman and Hermansson, 2015) used in Söderlund and Jobs (2016) in which they mimic more natural language processing and provide greater contextual support, which facilitates predictions of final words. Hagerman sentences on the other hand are based on a predictable grammatical structure (numeral + adjective + noun; e.g., *six new pencils*), but the listener cannot derive/infer the content prior to its being heard. In a recent review of the ELU model, Rönnberg et al. (2019) elucidated that SIN is less demanding upon working memory maintenance when the sentence materials are high on contextual support and lexical predictability. This addition to the ELU model offers an alternative explanation for the results in Söderlund and Jobs (2016). The use of Hagerman sentence materials may have prevented the possibility of a top-down advantage; i.e., the lack of contextual support may have impeded the possibility for children with more efficient/developed cognitive control to modulate the automaticity of inferential processes (c.f. Kiefer, 2007). Additionally, the axonal maturation in the superficial layers of the auditory cortex does not reach an equivalent density to that of adults until around 11 years (Moore and Linthicum, 2007). Thus, the demands placed on working memory maintenance by Hagerman sentences in combination with masking effects may have impacted both auditory and cognitive processing in these children to such a degree that there was very little variance between groups in the presence of noise (i.e., a flooring effect).

A second alternative is that the ADHD participants were able to perform on par with controls by exerting more effort in order to solve the task in working memory. In such a scenario, the ELU model predicts that individual differences in SRTs would correlate with individual differences in measures of complex working memory and cognitive load (Rönnberg et al., 2019). It is not reported in the pilot study (Söderlund and Jobs, 2016) whether SRT variance across participants in noise is negatively correlated with the measures of working memory and attention that demonstrated significant group differences (ADHD < Controls). Nonetheless, the discrepancy in results between studies indicates that a far more detailed and controlled experimental design is necessary to provide conclusive evidence for beneficial suprathreshold stochastic resonance effects as opposed to negative masking effects for individuals with ADHD in the domain of SIN processing. Furthermore, the present group is more heterogenous with regard to age than earlier studies on noise benefit (Söderlund et al., 2007, 2016; Helps et al., 2014), so developmental differences across children with ADHD must also be considered.

### Effect of Cognitive Capacity

A principal hypothesis of the ELU-model is that measures of cognitive capacity predict general SIN ability (Rönnberg et al., 2013). Multiple regression analysis was used to test this hypothesis in which capacity measures of cognitive control (i.e., combined proficiency in selective attention, working memory maintenance and inhibition) and lexical access efficacy were predictors, and participant’s overall HINT performance was the outcome variable. In addition, measures of hearing acuity in quiet were included in the model to see if individual differences at baseline could account for some of the variance in noise. All predictors had a significant association with HINT performance and together accounted for 69% of the variance. Importantly, although participants’ baseline hearing acuity was a contributing predictor of outcomes, individual differences in cognitive control and lexical access efficacy proved to be far stronger determinants of SIN ability. In addition, the two cognitive regressors were shown to contribute to relatively equal importance to model predictions. These findings robustly support the ELU model, which underscores the crucial involvement of top-down mechanisms when understanding speech in adverse listening conditions.

### Implications

The results of the current study have implications for our understanding of suitable classroom environments, and the types of solutions schools can employ to reduce listening effort in students with deficient cognitive capacity. Indeed, we have provided supportive evidence for the preliminary work of Schafer et al. (2013) who investigated whether normal-hearing children with autism spectrum disorder and ADHD could benefit from personal FM systems (frequency modulation systems) in the classroom. Their research utilized FM systems, which consisted of a small signal receiver fitted in the ear of the child that was paired with a microphone worn by the teacher and was designed to improve the SNR at the child’s ear without impeding sound stimulation from the natural environment. Schafer et al. (2013) noted that fitting autism spectrum disorder and ADHD participants with FM systems improved both SIN ability and listening behaviors in the classroom. Given that the current study observed that persons with ADHD required higher SNRs in noise conditions that are typical of classroom environments (e.g., background chatter, ventilation/fan noise), the use of personal FM systems (or other devices that can improve listening conditions by enhancing SNRs) may circumvent various behavioral problems associated with increased listening effort such as fatigue, distraction, and poor retention of information (cf. Peelle, 2018).

Additionally, the use of central stimulant medication has also been shown to improve both auditory processing and the subjective experience of listening effort and background noise disturbance in persons with ADHD (Keith and Engineer, 1991; Freyaldenhoven et al., 2005; Lanzetta-Valdo et al., 2016). Although the use of stimulant medication in children is controversial and the long-term health risks are still being investigated (c.f. Curtin et al., 2018), our findings together with previous reports provide reasons to consider the need for stimulant medication as a means to improve cognitive performance and facilitate learning in school-aged children with top-down processing deficits.

Another implication from our results offers potential improvements to diagnostic procedures in relation to ADHD. The consistently poorer performance observed in the ADHD group, along with mounting reports that persons with ADHD generally demonstrate inferior performance to controls on auditory processing tasks, suggests that SIN tests may prove to be beneficial clinical instruments when diagnosing ADHD. In particular, we have shown that the sound stimuli can manipulate cognitive load without the confounds of additional conceptual processing that is frequently involved in other popular neuropsychological measures of cognitive control. For instance, numerous working memory tasks require mathematical abilities or a developed concept of ordinals/seriality (e.g., the Paced Auditory Serial Addition Test, Operation-Span Task and Digit-Span Memory Task). By utilizing auditory conditions designed to tax attention and working memory capacity (i.e., noise and signal-quality manipulations) together with easily processed information (i.e., highly familiar speech materials), SIN tasks may aid diagnosticians in identifying deficits specific to top-down processing. Further research in this regard is therefore encouraged.

### Limitations

This study has several limitations. First, data collection had limited control over the test environment. Voluntary participants were recruited regardless of where they resided in Sweden. This entailed that the test leader travels to the participant and conducts testing in locations that were readily available to the participant. Even though care was taken to assure that the immediate environment was sufficiently quiet and isolated so as not interfere/override the stimuli presented in the headphones during testing, a controlled environment such as a soundproof lab, would have been more optimal. Second, the research was limited by a small sample size. Although we did aim to have a larger sample size, the actual response rate, particularly for the ADHD group, was much lower than anticipated for the time constraints of the project. A much larger sample would have allowed us to utilize more sophisticated analysis techniques (e.g., mixed modeling) and to pose more explorative questions about how the cognitive variables interact with individual participant’s performance in speech understanding. Third, only a scarce number of studies have researched the effects of background noise upon speech understanding in ADHD and they vary considerably in the types of test protocols employed (including speech and noise materials) and sample (i.e., age, gender, and sample size). This heterogeneity across studies renders comparisons of findings difficult. For these reasons, we refrained from postulating *a priori* hypotheses regarding the effects of specific noise types in our ADHD group and instead chose to explore several possible outcomes (O1–O3) based upon previous reports. Having observed consistently poorer performance for SIN in our sample of ADHD participants and noting that performance improved as a function of cognitive capacity across individuals, we argue for the necessity of replicative studies in order to refine our understanding of the extent in which deficits in attention and working memory impact speech processing.

## Conclusion

To conclude, the large body of research that forms the ELU model emphasizes the important contribution of top-down mechanisms when listening to speech in adverse conditions. To test this assumption more thoroughly, the current study investigated whether processing SIN is more difficult for normal-hearing adolescents diagnosed with ADHD than their age-matched counterparts. Our results showed that ADHD participants had greater difficulty than controls at listening to clear and degraded speech – both in noise and in quiet. In addition, individual differences in cognitive capacity greatly determined participants’ proficiency with understanding SIN. These findings provide additional support for the ELU model and further highlight the general disadvantage persons with deficient cognitive capacity have when attending to speech under challenging conditions.

## Data Availability

The datasets generated for this study are available on request to the corresponding author.

## Ethics Statement

This study was carried out in accordance with the recommendations of the Regional Ethics Committee in Linköping, Sweden. All participating minors were required to make an informed decision about participation on one’s own accord and their parents/guardians gave written informed consent in accordance with the Declaration of Helsinki. The protocol was approved by the Regional Ethics Committee in Linköping, Sweden (Dnr 2016/169-31).

## Author Contributions

JR, HD, and MR contributed to the conception and design of the study. RB was responsible for data collection, statistical analysis, drafting, and finalization of the manuscript. JR, HD, MR, and GS contributed to manuscript revision and approved the submitted version.

### Conflict of Interest Statement

The authors declare that the research was conducted in the absence of any commercial or financial relationships that could be construed as a potential conflict of interest.

## Abbreviations

ADHD: Attention deficit hyperactivity disorder
2BAB: Two-talker babble
CLR: Clear (non-distorted)
DSM: Diagnostic and Statistical Manual of Mental Disorders
ELU: Ease of language understanding
HINT: Hearing in noise task
MBA: Moderate brain arousal
NV: Noise vocoded
SIC-span: Size comparison span
SIN: Speech in noise
SNR: Signal-to-noise ratio
SRT: Speech reception threshold
SSN: Speech-shaped noise
WN: White noise.
